# Prognostic value of four immune-related genes in lower-grade gliomas: a biomarker discovery study

**DOI:** 10.3389/fgene.2024.1403587

**Published:** 2024-08-12

**Authors:** Shuowen Wang, Zijun Wang, Zhuo Liu, Jianxin Wu

**Affiliations:** ^1^ Capital Institute of Pediatrics, Beijing, China; ^2^ Beijing Tongren Hospital, Capital Medical University, Beijing, China; ^3^ Beijing Shijitan Hospital, Capital Medical University, Beijing, China

**Keywords:** lower-grade gliomas, TCGA, GTEx, immune-related genes, Chinese Glioma Genome Atlas (CGGA)

## Abstract

**Introduction:**

The tumor microenvironment and IRGs are highly correlated with tumor occurrence, progression, and prognosis. However, their roles in grade II and III gliomas, termed LGGs in this study, remain to be fully elucidated. Our research aims to develop immune-related features for risk stratification and prognosis prediction in LGG.

**Methods:**

Using the ssGSEA method, we assessed the immune characteristics of the LGG population. We conducted differential analysis using LGG samples from the TCGA database and normal samples from GTEx, identifying 412 differentially expressed immune-related genes (DEIRGs). Subsequently, we utilized univariate Cox, LASSO, and multivariate Cox regression analyses to establish both a gene predictive model and a nomogram predictive model.

**Results:**

Here, we found that the ESTIMATE score, immune score and stromal score of high-immunity, high-grade and isocitrate dehydrogenase (IDH) wild-type glioma were higher than those of the corresponding group, and the tumor purity was lower. Higher ESTIMATE scores, stromal scores and immune scores indicated a poor prognosis in patients with LGG. Our four-gene prognostic model demonstrated superior accuracy compared to other molecular features. Validation using the CGGA as a testing set and the combined TCGA and CGGA cohort confirmed its robust prognostic value. Additionally, a nomogram integrating the prognostic model and clinical variables showed enhanced predictive capability.

**Discussion:**

Our study highlights the prognostic significance of the identified four DEIRGs (KLRC3, MR1, PDIA2, and RFXAP) in LGG patients. The predictive model and nomogram developed herein offer valuable tools for personalized treatment strategies in LGG. Future research should focus on further validating these findings and exploring the functional roles of these DEIRGs within the LGG tumor microenvironment.

## 1 Introduction

Gliomas, including astrocytomas, oligodendrogliomas, and oligoastrocytomas, are the predominant malignant primary tumors that occur within the central nervous system of adults (Osuka and Van Meir, 2017; Jang and Kim, 2018). As per the 2021 fifth edition of the WHO Classification of Tumors of the Central Nervous System, adult diffuse gliomas are categorized as “astrocytoma, IDH-mutant,” “oligodendroglioma, IDH-mutant, 1p19q-codeletion,” or “glioblastoma, IDH-wildtype” (Louis et al., 2021). Typically, the aforementioned first two categories, “astrocytoma, IDH-mutant,” and “oligodendroglioma, IDH-mutant, 1p19q-codeletion,” are classified as “low-grade gliomas,” whereas glioblastomas are categorized as “high-grade gliomas” (Louis et al., 2021; Li et al., 2022). Importantly, The Cancer Genome Atlas (TCGA) program recognized the significantly higher malignancy of glioblastoma (grade IV glioma) and defined grade II and grade III gliomas as lower-grade gliomas (LGGs). This distinction diverged from the conventional definition of low-grade glioma based on the WHO classification.

LGGs account for approximately fifteen percent of all primary brain tumors in adults (Rasmussen et al., 2017; Berntsson et al., 2018; Li and Meng, 2019). In comparison to patients with glioblastomas, patients with LGGs generally have a more favorable prognosis (Claus et al., 2015). Nonetheless, importantly, the majority of individuals diagnosed with LGGs will eventually experience disease progression, transitioning to higher-grade gliomas, which ultimately result in mortality (Kiran et al., 2019). By conducting extensive long-term monitoring and collecting comprehensive demographic and clinical information, it becomes feasible to meticulously investigate the prognostic and predictive importance of pertinent genetic biomarkers. Significant attention has been directed toward IDH1/2 mutations because these markers play a fundamental role in the classification of gliomas according to the revised 2016 WHO CNS guidelines (Eckel-Passow et al., 2015; Louis et al., 2016). In recent years, there has been growing importance placed on molecular biomarkers, which serve as valuable adjuncts in providing diagnostic information and guiding treatment decisions. The World Health Organization (WHO) Classification of CNS tumors, specifically CNS5, has even directly incorporated genetic modifiers, such as IDH mutations and 1p/19q codeletion, into the nomenclature of gliomas, highlighting their crucial role in the diagnosis and classification of these tumors (Louis et al., 2021).

Significant advancements have been achieved in the management of LGGs, such as employing extensive surgical resection, radiotherapy, and chemotherapy. However, it remains common for these tumors to recur and develop drug resistance (Osuka and Van Meir, 2017). Thus, there is an urgent need to discover innovative biomarkers that can elucidate the underlying pathological mechanisms of LGG and facilitate the development of targeted therapeutic approaches for its treatment.

The role of the immune system in influencing cancer progression has been a research hotspot of research for over a century. Immunotherapy and immune checkpoint inhibitors have emerged as groundbreaking approaches to impede tumor growth by mitigating the immune evasion mechanisms employed by cancer cells (Harris and Kranz, 2016; Banchereau and Palucka, 2018). Notably, immune checkpoint inhibitors have garnered significant interest as a comprehensive and potent form of immunotherapy. These inhibitors function by obstructing inhibitory immune checkpoint pathways, thereby reactivating the antitumor immune response. For example, inhibitors targeting the PD-1/PD-L1 pathway have demonstrated remarkable efficacy in the treatment of melanoma (Long et al., 2018; Wan and Ming, 2018). However, the outcomes of immunotherapy for glioma have thus far been unsatisfactory (McGranahan et al., 2019). Therefore, it is crucial to comprehend the immune mechanisms specific to LGGs and explore novel immune checkpoints that could offer a new avenue for immunotherapy.

Although previous studies have examined the relationship between immune-related genes (IRGs) and the prognosis of patients with LGGs, most of them have focused on the individual functions of single genes. Few studies have utilized high-throughput sequencing to explore the correlations between multiple immune genes and the prognosis of LGG. In this study, we aimed to address this gap by employing the single-sample gene set enrichment analysis (ssGSEA) method to classify LGG patients from TCGA and CGGA databases into two distinct clusters: the immune-high and immune-low clusters. To validate the molecular and immune patterns of these clusters, we employed the ESTIMATE algorithm. Furthermore, a combination of least absolute shrinkage and selection operator (LASSO) regression and Cox regression analysis was applied to construct an IRG prognostic model. Finally, we combined clinical information with the prognostic model to create a nomogram to improve the prediction of the 1-year, 3-year, and 5-year overall survival rates of LGG patients.

## 2 Materials and methods

### 2.1 Cell line

This study utilized two distinct cell lines, SVGp12 and U-373 MG, purchased from YaJi Biological (Shanghai, China). SVGp12 cell line, derived from normal human astrocytes obtained from the human brain, was used as the control group to represent normal cellular characteristics. SVGp12 cells were cultured in complete DMEM medium composed of 89% DMEM, 10% fetal bovine serum (FBS), and 1% penicillin-streptomycin (PS).

On the other hand, the U-373 MG cell line, derived from human glioblastoma multiforme, served as the experimental group, representing glioma cell characteristics. U-373 MG cells were maintained under the same standard cell culture conditions, using complete DMEM medium composed of 89% DMEM, 10% FBS, and 1% PS. Both cell lines were cultured in a sterile environment at 37°C with 5% CO_2_ incubation.

### 2.2 Databases

The transcriptomic data and clinical information for LGG and glioblastoma (GBM) patients were downloaded from the TCGA portal (https://portal.gdc.cancer.gov/). The molecular data, along with clustering and subtyping information of TCGA samples, were extracted from the Supplementary Material of previous studies (Ceccarelli et al., 2016). To obtain a reference for normal samples, the transcriptomic data of 1,152 normal samples were downloaded from GTEx (https://gtexportal.org/home/datasets). The RNA-seq data from the CGGA database (https://www.cgga.org.cn), along with corresponding clinical information, were utilized to validate the risk model. A comprehensive list of IRGs was obtained from the ImmPort database (https://www.immport.org/home), which serves as a valuable resource for immunology research. The ID numbers of the patient transcriptomic data were matched with the patient OS data, age data, IDH mutation data, and grading data. Any data entries that did not have matching ID numbers were removed from the dataset. Consequently, we obtained transcriptomic and clinical information from 500 LGG patients in the TCGA database and 552 LGG patients in the CGGA database, ensuring that the dataset was complete. Information regarding immune infiltrates of six types of immune cells (B cells, CD4^+^ T cells, CD8^+^ T cells, neutrophils, macrophages, and dendritic cells) was acquired from the TIMER database (http://www.cistrome.org/). Our access to all databases was concentrated in November 2022.

To process the data, R software (https://www.r-project.org/) was used. To mitigate the impact of batch effects, we employed the “limma” package embedded within R to normalize the combined transcriptomic data from different databases.

For the benefit of a broader audience with varying backgrounds, we have created a graphical abstract (Supplementary Figure Graphical Abstract).

### 2.3 Clustering of the LGG patients

The ssGSEA method is a recently introduced algorithm designed to quantify immune cell subsets using RNA samples obtained from diverse tissue types, including solid tumors (Bindea et al., 2013). In this particular study, the ssGSEA method was employed to determine the absolute enrichment fraction of 29 immune cells, along with their immune-related functions and pathway marker genes, in patients with LGG. To further analyze the LGG samples from the TCGA and CGGA databases, the R package “GSVA” was utilized for computing the ssGSEA score levels for gene sets in LGG samples. Following, we utilized the SPARCL algorithm provided by the “sparcl” package to conduct clustering analysis, thereby clustering the samples into two distinct groups: immune-high and immune-low. These approach enabled the classification of samples based on their levels of immune activity and allowed for a deeper understanding of their immune characteristics within the context of LGG.

### 2.4 Survival analysis

Kaplan-Meier analysis coupled with the log-rank test was performed to identify IRGs with prognostic significance. The “Survival” and “Survminer” packages in R were utilized to visualize the associations between gene expression levels and the outcomes of patients with LGG by plotting survival curves.

### 2.5 Evaluating the efficacy of immune clustering, IDH mutations and grade

To evaluate the effectiveness of ssGSEA clustering, IDH mutations, and grade, the ESTIMATE algorithm was employed. The R package “ESTIMATE” was used to calculate the ESTIMATE scores, immune scores, stromal scores, and tumor purity for each sample of LGG in different groups.

### 2.6 Identification of differentially expressed immune-related genes (DEIRGs)

The differential expression gene profile between normal samples in the GTEx database and LGG samples in the TCGA database was determined by the R package “limma” (FDR<0.05 and |log2 [FC]| > 1). The IRGs obtained from the ImmPort database were intersected with the IRGs to obtain DEIRGs. The “pheatmap” package is employed to generate heatmaps, where the default clustering coefficient is set to 0.75. This implies that elements with a similarity exceeding 0.75 are grouped into the same cluster.

### 2.7 Risk model construction

The clinical data of LGG samples from the TCGA dataset were analyzed using the “Survival” package in R through univariate Cox regression analysis. This analysis aimed to identify IRGs that exhibited a significant association with the survival of LGG patients.

To further refine the selection of prognostic genes, a LASSO analysis was performed using the “glmnet” package in R. Based on the training set, the optimal penalty coefficient (log(λ) = −3.4) was determined.This analysis helped identify candidate risk genes with the potential to predict patient outcomes in LGG.

The selected risk genes were then assessed for their role in prognosis using multivariate Cox regression analysis. Multivariate Cox regression considers multiple factors to determine the impact of each factor on survival time after adjusting for other factors.

To integrate the expression levels of the identified risk genes, a risk score for each patient was calculated. The risk score was calculated by summing the products of the gene expression level and its corresponding regression coefficient. This score provided a quantitative measure of the patient’s risk and could be used to stratify patients into different risk groups based on their predicted prognosis. The specific formula was as follows:

Risk score = 12.4241 × exp (−0.4939 × expression of KLRC3 + 0.2125 × expression of MR1 + −0.1655 × expression of PDIA2 + −0.4283 × expression of RFXAP).

### 2.8 Quantitative real-time PCR (qRT-PCR)

One microgram of RNA was reverse transcribed using HiScript II Q Select RT SuperMix for reverse transcriptase PCR, which includes a gDNA wiper (Vazyme, R323, China). The qRT-PCR was then conducted using the CFX96 Touch Real-Time PCR Detection System (Bio-Rad), employing ChamQTMSYBRqPCR Master Mix (Vazyme, Q711, China) along with the specified primers. The analysis of gene expression was carried out by the relative quantification 2^−ΔΔCT^ method, using 18S rRNA as the normalization control.

Human KLRC3 qPCR Primer Pair (Beyotime, QH17145S), Human PDIA2 qPCR Primer Pair (Beyotime, QH50201S), Human RFXAP qPCR Primer Pair (Beyotime, QH21601S), Human RNA18SN5 qPCR Primer Pair (Beyotime, QH00093S), Human MR1 qPCR Primer Pair (Forward Primer - TGG​GGT​CCC​TGA​ATT​TAT​TTC​G, Reverse Primer - TTC​CAC​CTT​GAA​CAT​CTG​CTG).

### 2.9 Immune cell infiltration analysis

The estimation of immune infiltration in LGG samples was performed using the TIMER and ssGSEA algorithms. These algorithms were utilized to assess the presence and abundance of immune cells within the tumor microenvironment. The TIMER algorithm, which incorporates six major types of immune cells, including B cells, CD4^+^ T cells, CD8^+^ T cells, dendritic cells, neutrophils, and macrophages, was employed to evaluate the relative abundance of each immune cell type.

### 2.10 Construction of the nomogram

A nomogram is a useful tool for predicting survival rates in LGG patients. In this study, the R packages “rms” (root mean square) and “survival” were utilized to develop a nomogram based on IRG prognostic markers. The risk score, age, IDH mutation status, and tumor grade were considered predictors in the nomogram model.

### 2.11 Statistical analyses

All statistical analyses were conducted using R software (version 4.0.2). A p-value <0.05 was considered to indicate statistical significance. The Pearson correlation coefficient test was employed to assess the rank correlation among variables. To determine the differences between variables, the independent t-test was utilized. For differential analysis and normalization, the “limma” package in R was employed. We utilized the “survival” package in R to conduct either Cox regression modeling or Kaplan-Meier analysis accompanied by a log-rank test. These analyses were employed to evaluate the associations between the identified prognostic risk factors and the survival outcomes of LGG patients.

To assess the accuracy of the prognostic risk model, time-dependent receiver operating characteristic (ROC) analysis was performed. Additionally, the concordance index (C-index) was calculated. These metrics were used to evaluate the predictive performance of the model.

In terms of the prediction values, an area under the curve (AUC) greater than 0.60 was considered acceptable, while an AUC greater than 0.70 was considered favorable. Similarly, a C-index greater than 0.60 was considered acceptable in evaluating the performance of the prognostic risk model.

## 3 Results

### 3.1 Construction and validation of LGG clustering

**FIGURE 1:**
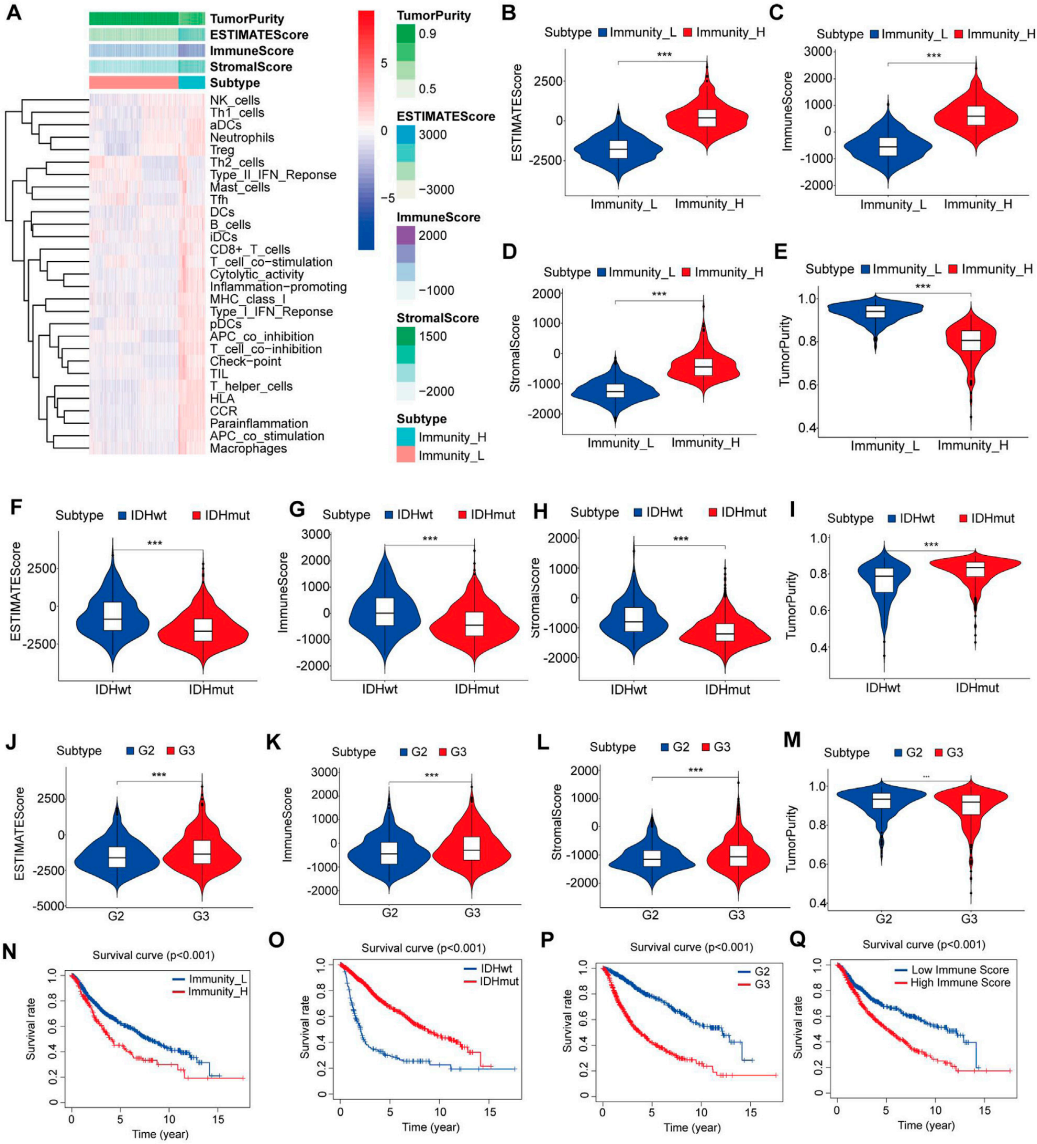
Construction and verification of LGG clustering. **(A)** The heatmap showed that the 29 immune-related cell types had high expression in the high-immune cell infiltration group (Immunity-high), and low expression in the low immune cell infiltration group (Immunity-low). The Tumor purity and ESTIMATE, Immune, and Stromal Scores of each patient are shown with clustering information using the ESTIMATE algorithm. **(B–E)** The violin plot shows the difference in the ESTIMATE, Immune, Stromal Scores and Tumor purity between the two clusters. **(F–I)** The violin plot shows the difference in the ESTIMATE, Immune, Stromal Scores and Tumor purity between the IDH mutant group and the IDH wild-type group. **(J–M)** The violin plot shows the difference in the ESTIMATE, Immune, Stromal Scores and Tumor purity between the grade II and III gliomas. **(N)** Kaplan–Meier curves for overall survival in the high and low immune cell infiltration groups. **(O)** Kaplan–Meier curves for overall survival in the IDH mutant and IDH wild type groups. **(P)** Kaplan–Meier curves for overall survival in the grade II and grade III gliomas. **(Q)** Kaplan–Meier curves for overall survival in the high and low immune‐score groups. Data are represented as mean ± SD. *p < 0.05; **p < 0.01; ***p < 0.0001.

The data from a cohort of 1152 LGG patients was collected from both the TCGA database and the CGGA database. The ssGSEA score was calculated for each LGG sample, and based on this score, the samples were divided into two clusters using an unsupervised hierarchical clustering algorithm (Figure 1A). To assess the reliability of the clustering results, we employed the ESTIMATE algorithm to calculate the tumor purity, stromal score, immune score, and ESTIMATE score based on the expression profiles of each LGG sample. The analysis revealed that the immune-high cluster group had significantly higher stromal, immune, and ESTIMATE scores and lower tumor purity scores than the immune-low cluster group (p < 0.05) (Figures 1B–E). Furthermore, through Kaplan-Meier analysis, we observed a significant correlation between immune clustering and the survival outcomes of LGG patients (p < 0.05) (Figure 1N).

We also found that IDH wild-type gliomas had significantly higher stromal, immune, and ESTIMATE scores and lower tumor purity scores than IDH mutant gliomas (p < 0.05) (Figures 1F–I). Additionally, grade III gliomas exhibited significantly higher stromal, immune, and ESTIMATE scores and lower tumor purity scores than grade II gliomas (p < 0.05) (Figures 1J–M).

Subsequently, we categorized the LGG patients into high- and low-score groups based on their immune scores, using the mean score as the cutoff value. Kaplan‒Meier survival analysis demonstrated a significant association between the immune score, IDH mutation status, and glioma grade and the survival of LGG patients (p < 0.05) (Figures 1O–Q). These findings suggest that the patient’s immune microenvironment may have comparable prognostic significance to IDH mutation status and tumor grade in LGG patients.

### 3.2 Identification of DEIRGs

To compare differences in gene expression patterns between normal brain samples from the GTEx database and LGG samples from the TCGA database, a significance threshold of [FDR] < 0.05 and |log2[FC]| > 1 was applied. This resulted in the identification of 8832 differentially expressed genes (DEGs), including 4358 upregulated genes and 4474 downregulated genes (Supplementary Figures S1A, B). Furthermore, we obtained 1793 IRGs from the ImmPort database. By integrating these IRGs with the DEGs, we selected 412 DEIRGs for further analysis using the criteria of [FDR] < 0.05, |log2[FC]| > 1 (Supplementary Figures S1C, D). These DEIRGs represent genes that are both differentially expressed and relevant to the immune response in LGG samples.

### 3.3 Identification of prognostic DEIRGs

**FIGURE 2:**
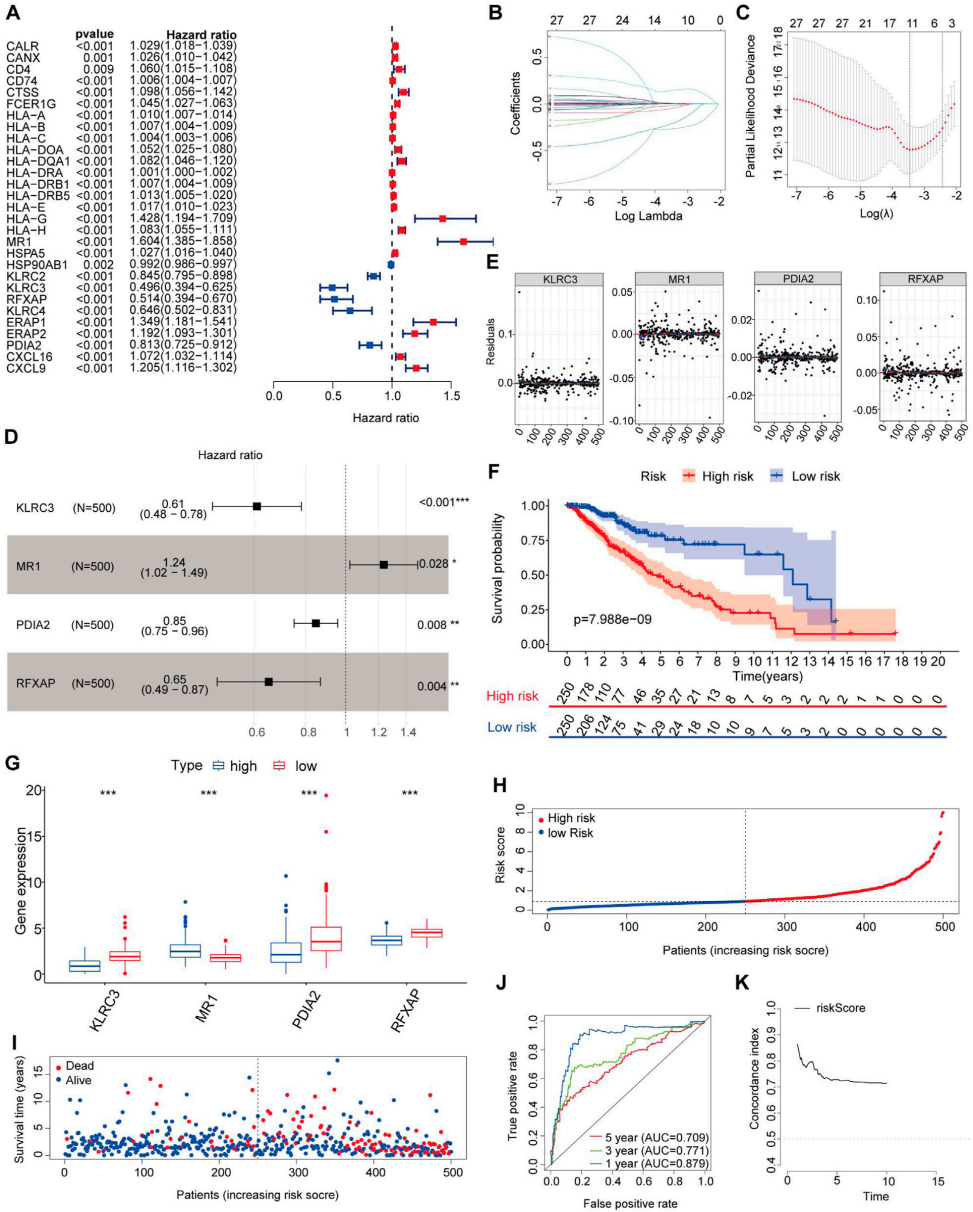
Risk genes in the prognostic risk model. **(A)** Using univariable Cox regression analysis, the HR and p-value for the chosen genes in the immune-related prognostic genes. **(B,C)** PDEIRGs selected through Lasso regression. **(D)** Multivariate Cox analysis identified four immune-related genes. **(E)** The Schoenfeld residual that assessed what factors can be incorporated into the model. **(F)** Kaplan-Meier curve analysis of the high-risk and low-risk groups. **(G)** Expression trend of risk genes in the prognostic model. **(H)** Risk score distribution of patients in the prognostic model. **(I)** Survival status scatter plots for patients in the prognostic model. **(J)** Time-dependent ROC curve analysis of the prognostic model. **(K)** Concordance index of the indicated prognostic model in the training set.

In our study, we utilized a training set comprising 500 LGG patients with complete clinical data for further analysis. As the immune-related signatures demonstrated differential levels between tumor and normal samples, we aimed to identify prognostic genes among them. To accomplish this, we performed univariate Cox regression analysis on all DEIRGs in the TCGA training set to identify potential prognostic DEIRGs (PDEIRGs). Our analysis revealed that 29 DEIRGs significantly influenced OS in LGG (p < 0.05) (Figure 2A).

Since the PDEIRGs had a significant impact on patient outcomes, we constructed a Cox regression hazards model by further refining the selection of PDEIRGs. To reduce the complexity of the prognostic signature, we employed Lasso regression to eliminate PDEIRGs that exhibited high correlation with each other. Following the assessment, the optimal penalty coefficient [log(λ)] was determined to be −3.4, corresponding to a scenario where 11 genes exhibited non-zero Coef values (Figures 2B, C).

Subsequently, we employed multivariate Cox regression model to calculate the regression coefficient for each gene. Through this rigorous selection process, we identified four optimal PDEIRGs, referred to as risk genes, for the prognostic risk model. These genes included KLRC3, MR1, PDIA2, and RFXAP. Among them, MR1 was found to be associated with a higher risk of death, predicting a poor prognosis. However, KLRC3, PDIA2, and RFXAP were associated with a lower risk of death, acting as protective factors (p < 0.05) (Figure 2D).

Furthermore, we plotted the Schoenfeld model residuals against the expression levels of KLRC3, MR1, PDIA2, and RFXAP. Based on this assessment, we preliminarily concluded that these predictive factors should be incorporated into the model (Figure 2E).

**FIGURE 3:**
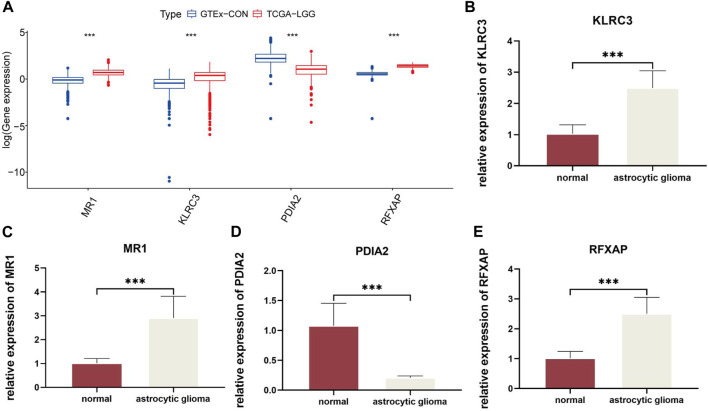
Quantitative analysis of risk genes using qRT-PCR: **(A)**. Expression of four risk genes in GTEx-CON and TCGA-LGG. **(B–E)**. Relative differences in gene expression of risk genes between normal and astrocytic glioma cell lines. In this figure, p< 0.05 was presented with “*”, p< 0.01 was presented with “**”, p< 0.001 was presented with “***”.

By comparing the normal and LGG samples from the database, we identified the expression trends of these four genes (Figure 3A). Subsequently, to ensure the reliability of risk genes, we referenced existing studies (Wu et al., 2023) and validated the expression of each risk gene through qRT-PCR experiments (Figures 3B–E). This suggests a significant association between immune-related risk genes and the onset of gliomas. Importantly, these risk genes serve as reliable biomarkers with potential clinical applicability.

### 3.4 Construction of the prognostic risk model of the four-gene signature

The regression coefficients of the selected PDEIRGs were utilized to construct a risk model, which allowed us to calculate a risk score for each patient. The formula was as follows:

Risk score = 12.4241 × exp (−0.4939 × expression of KLRC3 + 0.2125 × expression of MR1 + −0.1655 × expression of PDIA2 + −0.4283 × expression of RFXAP).

Utilizing the aforementioned equation, we calculated the risk score for each patient in the training set and arranged them in ascending order. Based on the median risk score (risk score = 0.9130), we divided the training set into two groups: a high-risk group (risk score >0.9130) consisting of 250 patients and a low-risk group (risk score <0.9130) consisting of 250 patients. To assess the associations between risk scores and OS, we performed Kaplan-Meier analysis with the log-rank test. This analysis revealed that patients with high-risk scores had significantly poorer OS than those with low-risk scores (p < 0.05) (Figure 2F).

Further examination of the high-risk training set revealed that the 3-year and 5-year OS rates were approximately 65.9% and 47.35%, respectively. In contrast, the corresponding OS rates in the low-risk training set were approximately 86.4% and 75.5%, respectively. To determine the expression patterns of the four genes used in the risk model, we generated a heatmap comparing their expression levels between the high-risk and low-risk groups. The high-risk group exhibited higher expression levels of the high-risk gene (MR1) and lower expression levels of the protective genes (KLRC3, PDIA2, and RFXAPP) (Figure 2G). Conversely, the expression pattern in the low-risk group showed the opposite trend.

Figures 2H, I present the distribution of survival risk scores and the dot plot of survival status for each patient in the training set, respectively. To evaluate the prognostic accuracy of the risk score, we employed time-dependent receiver operating characteristic (ROC) curves and calculated the corresponding area under the curve (AUC) values. The AUC values for the 1-year, 3-year, and 5-year predictions were approximately 0.879, 0.771, and 0.709, respectively (Figure 2J).

To assess the performance of our risk score model, we computed the C-index in the training set for each year from the first to the 10th. The C-index values obtained were greater than 0.6, indicating a reasonable discriminatory capacity of the risk score model (Figure 2K).

**FIGURE 4:**
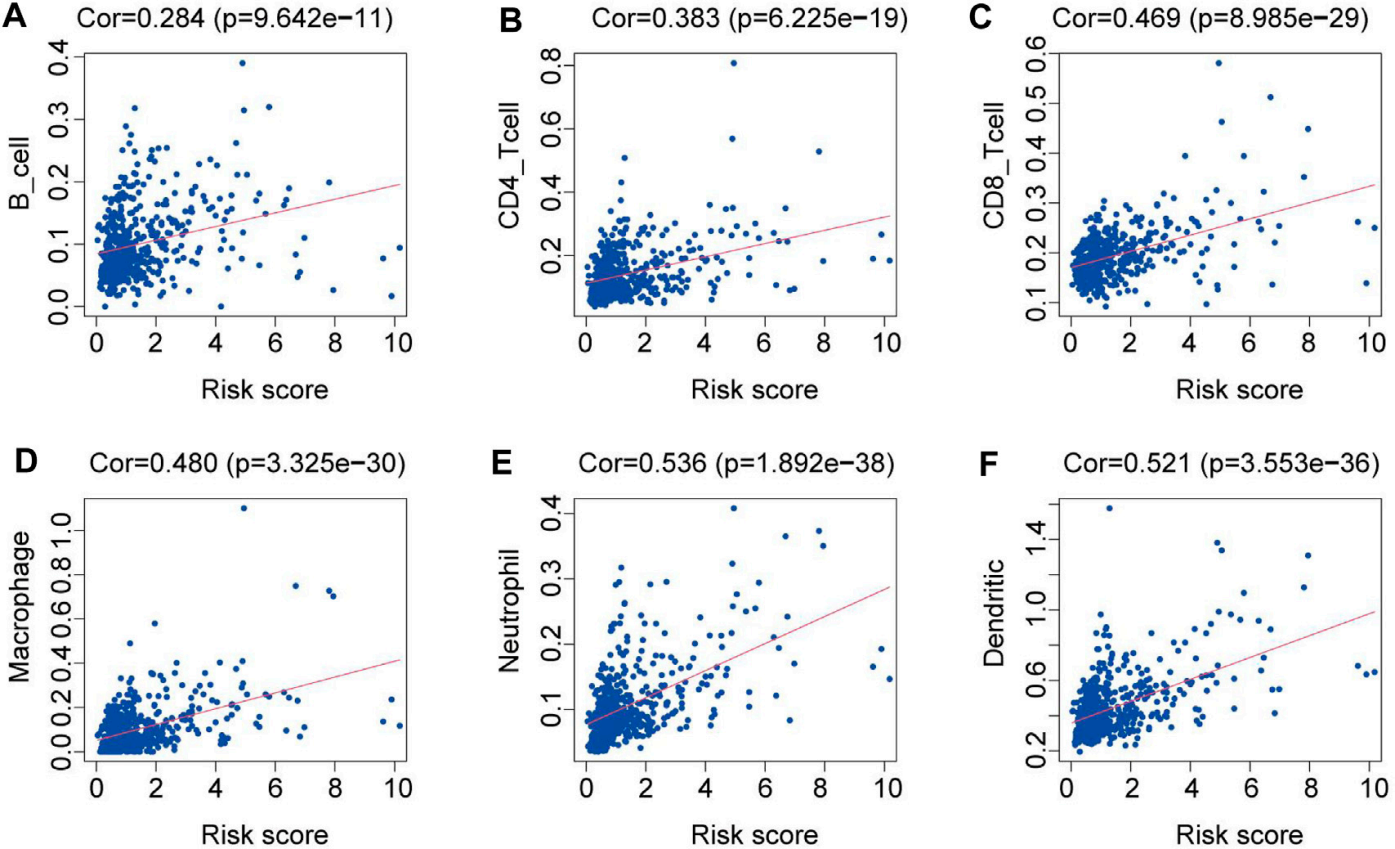
Relationships between the risk score and the abundance of immune cell infiltration. **(A)** B cells. **(B)** CD4 T cells. **(C)** CD8 T cells. **(D)** Macrophages. **(E)** Neutrophils. **(F)** Dendritic cells.

By examining the relationship between the proportion of immune cell types within tumor samples from the TCGA dataset and the risk scores, we identified a significant correlation. Specifically, we found that patients with higher risk scores tended to exhibit increased levels of immune cell infiltration, including those of B cells, CD4^+^ T cells, CD8^+^ T cells, neutrophils, macrophages, and dendritic cells (p < 0.05). This correlation is illustrated in Figures 4A–F.

### 3.5 Assessment of the reliability of the risk model

**FIGURE 5:**
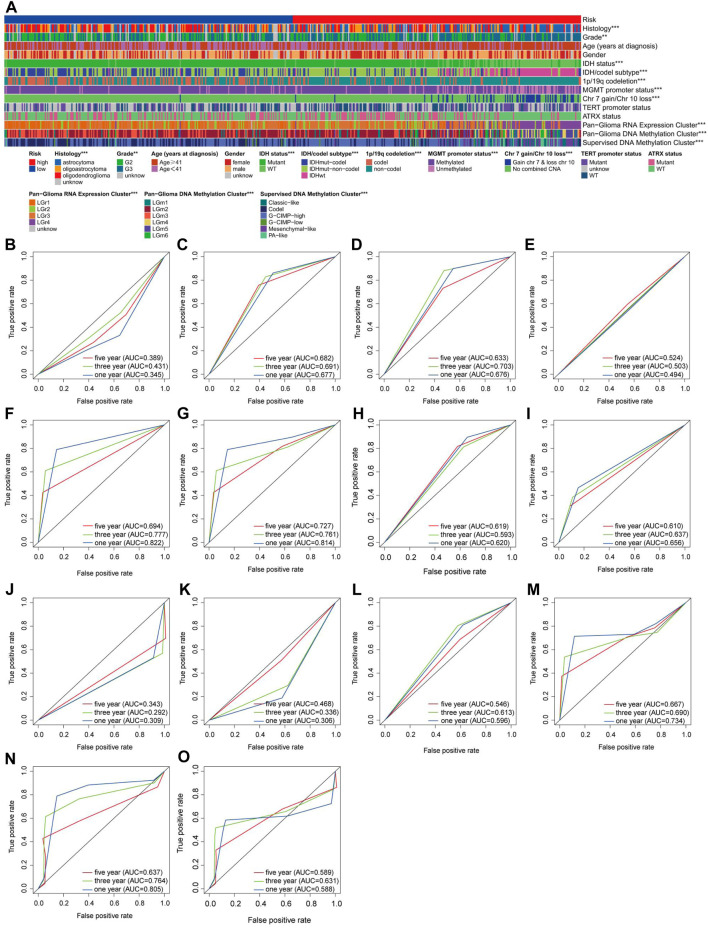
Assessment of the reliability of the risk model **(A)** A overall review of the differential distribution of molecular spectra between two risk subtypes. **(B–O)** 1-, 3- and 5-year OS-dependent ROC curves for various molecular subtypes in the TCGA cohort. Histology ROC **(B)**, Grade ROC **(C)**, Age (years at diagnosis) ROC **(D)**, Gender ROC **(E)**, IDH status **(F)**, IDH/codel subtype ROC **(G)**, 1p/19q codeletion ROC **(H)**, MGMT promoter status ROC **(I)**, Chr 7 gain/Chr 10 loss ROC **(J)**, TERT promoter status ROC **(K)**, ATRX status ROC **(L)**, Pan-Glioma RNA Expression Cluster ROC **(M)**, Pan-Glioma DNA Methylation Cluster ROC **(N)**, Supervised DNA Methylation Cluster ROC **(O)**.

Previous studies have demonstrated that clinically relevant molecular subtypes, such as IDH mutation, 1p/19q co-deletion status, and DNA methylation profiles, provide valuable prognostic indications for LGG (Ceccarelli et al., 2016). We observed extensive molecular profile heterogeneity between these two subtypes in our risk model, which has significant implications for assessing its reliability (Figure 5A). The vast majority of molecular subtypes exhibited significant differences between the two risk subtypes, indicating that our risk model provides valuable prognostic insights for LGG. To further demonstrate the superiority of our model, we assessed the performance of various molecular subtype classifications using ROC curves (Figures 5B–O). Overall, only the classification models for IDH status, IDH/codel subtypes, pan-glioma RNA expression cluster, and pan-glioma DNA methylation cluster demonstrated excellent performance (AUC>0.7) (Figures 5F, G, M, N). However, in terms of predicting accuracy for 1-year, 3-year, and 5-year outcomes, they still lagged behind our risk score model (Figure 2J).

### 3.6 The performance of the prognostic model

In the CGGA database, we utilized a cohort of 552 eligible LGG patients as the external testing set to evaluate the validity and accuracy of our prognostic risk model. Additionally, we combined the TCGA and CGGA datasets to create an expanded cohort with a total of 1052 patients and applied batch correction to ensure robust validation. Based on the median value of risk scores calculated from the training set, both the testing set and the entire set of LGG patients were divided into high-risk (n = 349 and n = 599, respectively) and low-risk (n = 203 and n = 453, respectively) groups.

**FIGURE 6:**
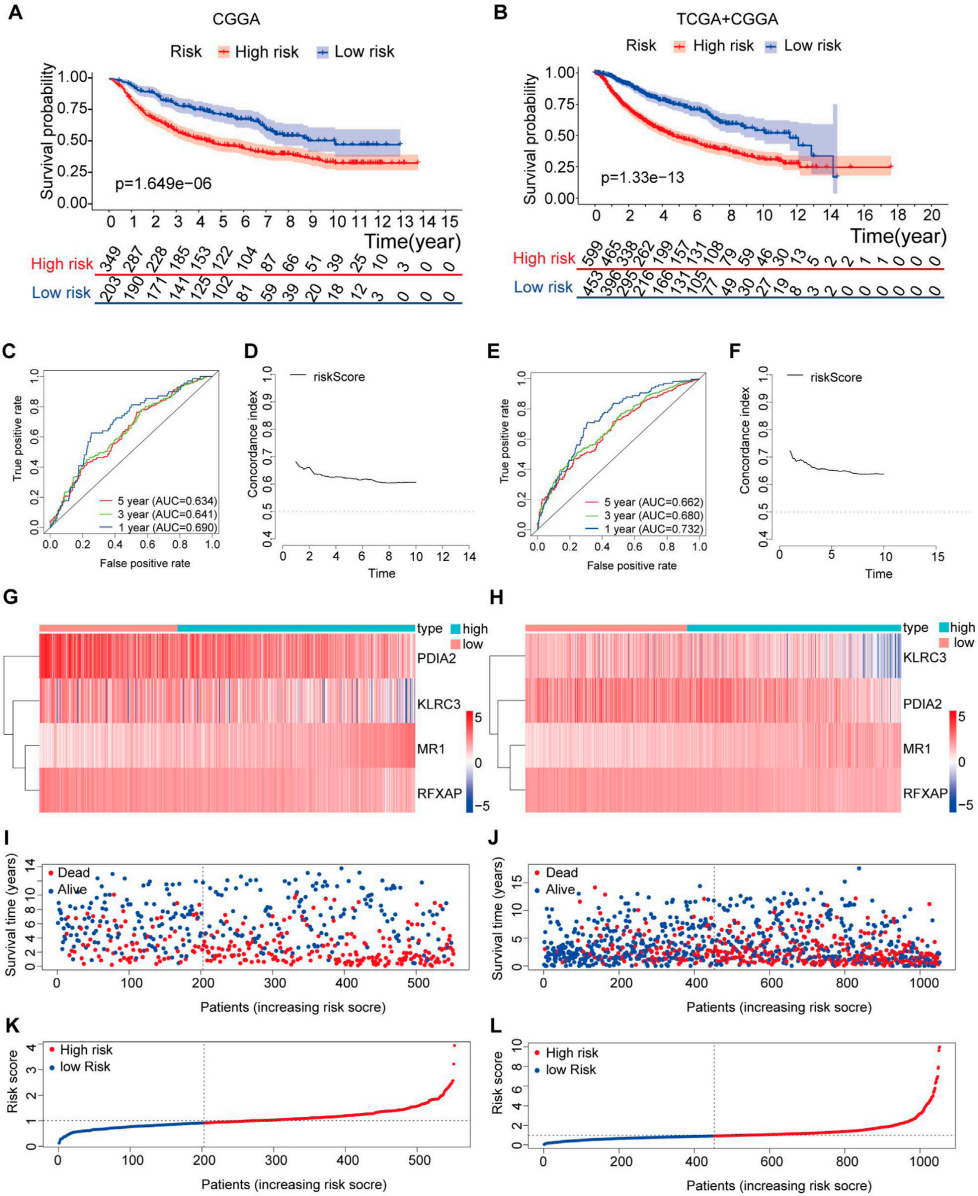
Validation of the immune-related gene prognostic signature in the testing and entire sets. **(A,B)** The survival status of patients in the high-risk and low-risk groups in the testing and entire sets. **(C)** The prognostic signature’s time-independent ROC curve at 1-, 3-, and 5-year in the testing. **(D)** Concordance index of the indicated prognostic model in the testing set. **(E)** The prognostic signature’s time-independent ROC curve at 1-, 3-, and 5-year in the entire set. **(F)** Concordance index of the indicated prognostic model in the entire set. **(G–H)** Expression patterns of risk genes in the testing and entire sets. **(I,J)** A scatter plot depicts the survival of LGG samples in the testing and entire sets. **(K,L)** Each LGG sample’s risk curve is reordered by the risk score in the testing and entire sets.

The Kaplan‒Meier survival analysis conducted in both the testing set and the entire set revealed a significant improvement in OS in the low-risk group compared to the high-risk group (p < 0.05) (Figures 6A, B). Specifically, in the testing set, the 3-year and 5-year survival rates were 58.3% and 46.6% in the high-risk group, while those in the low-risk group were higher at 78.8% and 70.7%, respectively (Figure 6A). These findings were further supported by the area under the curve (AUC) analysis, where the 1-year, 3-year, and 5-year AUCs were 0.690, 0.641, and 0.634, respectively, indicating a high sensitivity and specificity of our proposed risk model (Figure 6C).

Similarly, in the entire set, the 3-year and 5-year survival rates were 61.3% and 48.0% in the high-risk group, while those in the low-risk group were higher at 82.8% and 73.9%, respectively (Figure 6B). The AUC values at 1 year, 3 years, and 5 years were 0.732, 0.680, and 0.662, respectively, further confirming the accuracy of our risk model in predicting patient outcomes (Figure 6E).

To assess the performance of our risk score model over time, we calculated the C-index from the first year to the 10th year in both the testing set and the entire set, with values exceeding 0.6 (Figures 6D, F). These results demonstrate that our risk model, based on four specific genes, exhibits high sensitivity and specificity, making it a reliable predictor of OS in LGG patients.

Figures 6G–L present the distribution of risk scores, survival status, and gene expression levels in the testing set and the entire set. The findings were consistent with the training set, where the low-risk group exhibited higher expression levels of protective genes and lower expression levels of risk genes than the high-risk group.

**FIGURE 7:**
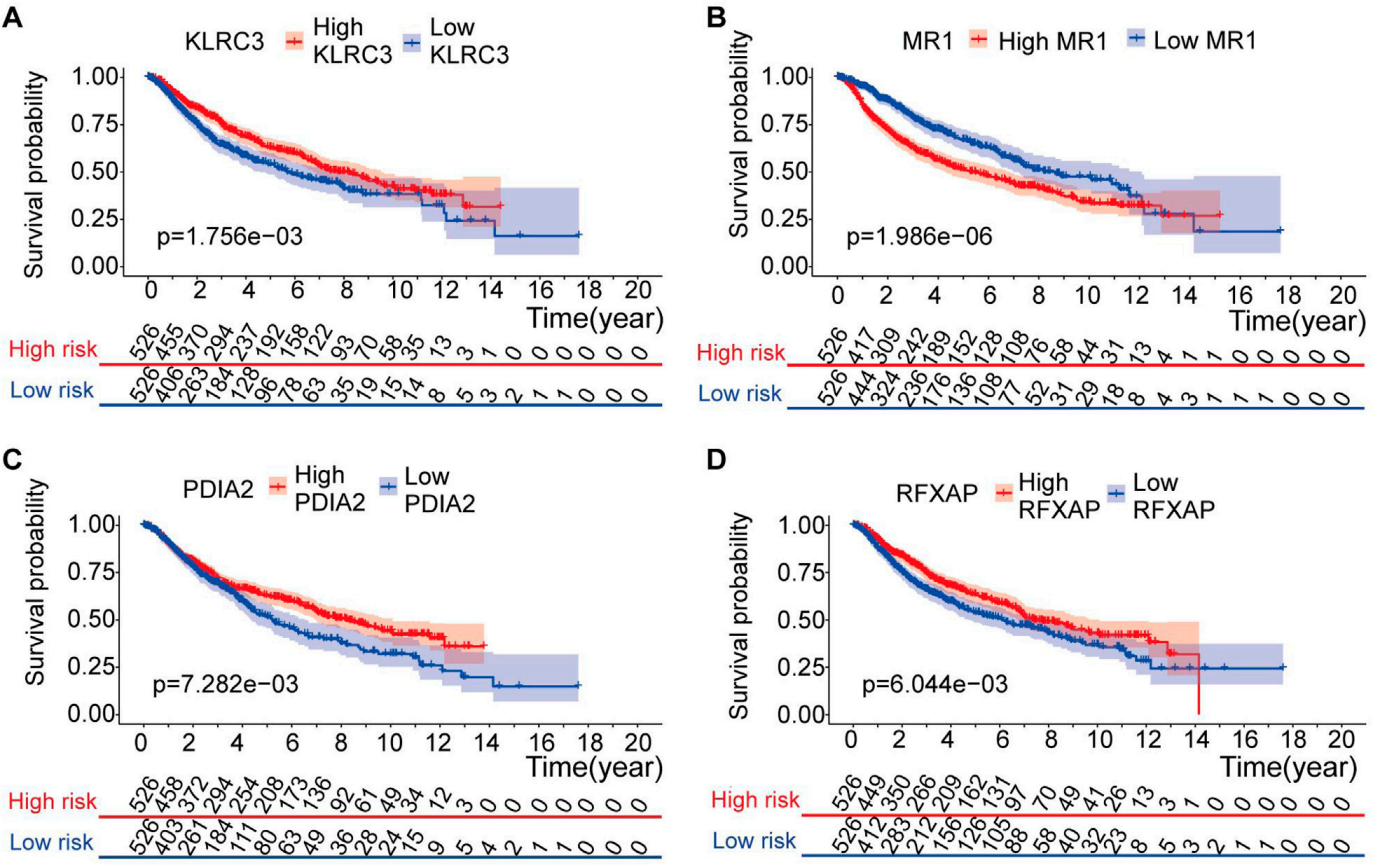
Kaplan-Meier analysis of risk model genes in the entire set. **(A–D)** Kaplan-Meier analysis of KLRC3 value **(A)**, MR1 value **(B)**, PDIA2 value **(C)** and PLEKHA4 value **(D)** in patients with LGG.

Furthermore, the four genes included in the risk model (KLRC3, PDIA2, RFXAP, and MR1) showed a significant correlation with the OS rate of LGG patients (p < 0.05) (Figures 7A–D). Specifically, higher expression levels of KLRC3, PDIA2, and RFXAP and lower expression levels of MR1 were associated with a relatively favorable prognosis in LGG patients.

### 3.7 Evaluation of IRG prognostic markers as independent prognostic factors

**FIGURE 8:**
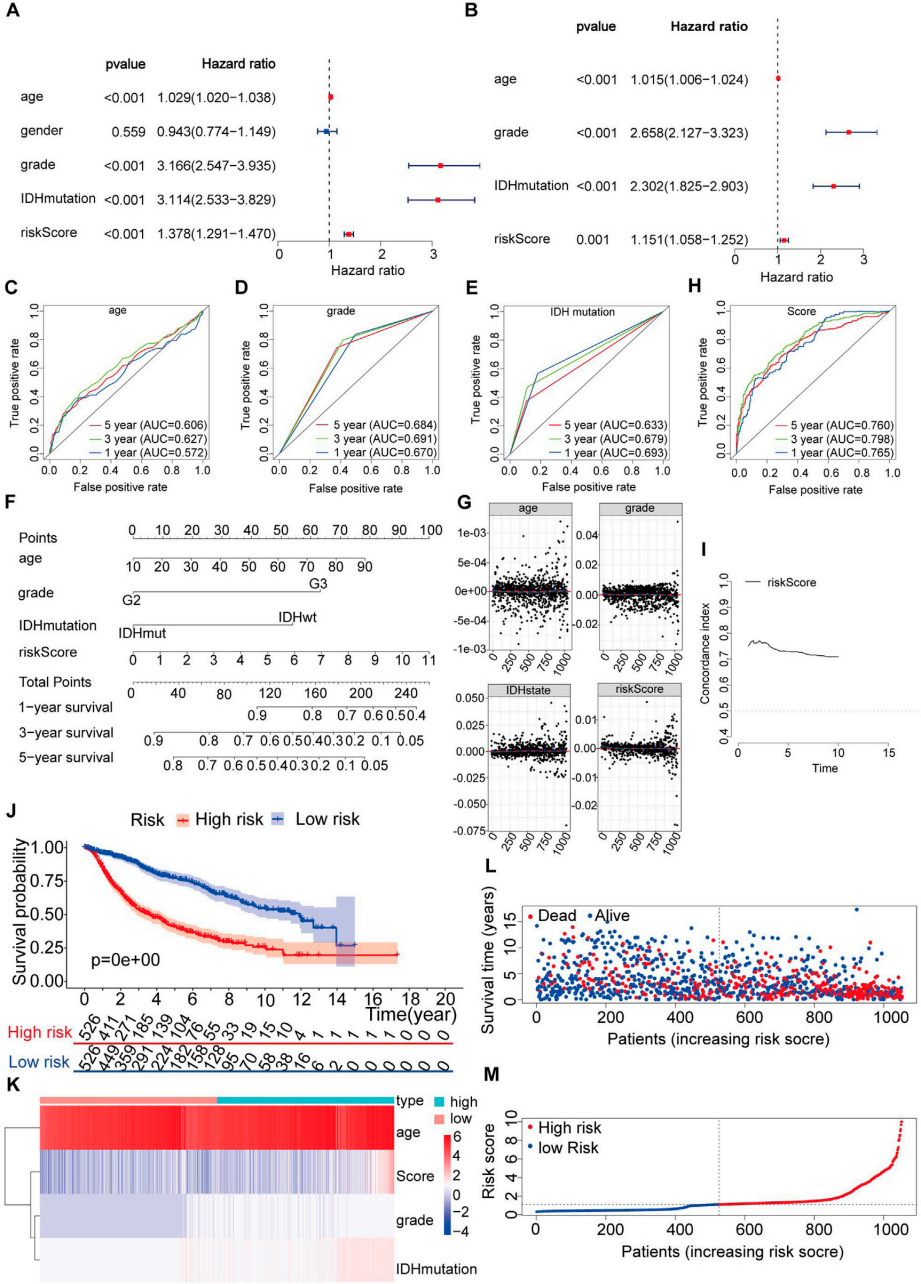
Construction of a nomogram and verification that the immune-related gene prognostic signature is an independent prognostic factor. **(A,B)** Univariate and multivariate Cox regression analysis of the immune-related gene prognostic signature in LGG patients to determine independent risk variables. **(C–E)** The combined ROC for age, grade and IDH mutation at 1-, 3-, and 5-years. **(F)** The development of a nomogram based on the immune-related gene prognostic signature in the entire set. **(G)** The Schoenfeld residual that assessed what factors can be incorporated into the nomogram model. **(H)** The combined ROC for nomogram at 1-, 3-, and 5-years. **(I)** Concordance index of the indicated nomogram prognostic model in the entire set. **(J)** Kaplan-Meier curve analysis of the high-risk and low-risk groups. **(K)** Expression patterns of risk factors in the nomogram prognostic model. **(L)** Survival status scatter plots for patients in the nomogram prognostic model. **(M)** Risk score distribution of patients in the nomogram prognostic model.

In the subsequent analysis, univariate Cox analysis and multivariate Cox analysis were performed to evaluate whether the risk score could serve as an independent prognostic indicator in addition to other clinical parameters such as age, sex, grade, and IDH mutation status for LGG patients. Both univariate and multivariate Cox analyses revealed significant associations between age, grade, IDH mutation status, risk score, and OS (p < 0.05) (Figures 8A, B). Since IDH mutation status is a crucial prognostic indicator designated by the World Health Organization (WHO) for LGG, the entire dataset was further divided into mutant and wild-type IDH groups. Subsequently, the risk model was utilized to assess whether the risk prediction could be made independent of IDH status (Supplementary Figure S2). The results indicated that the risk model could indeed be employed as an independent predictor for predicting the prognosis of LGG patients, regardless of their IDH mutation status.

The accurate grading of LGG is pivotal for precise diagnosis, treatment decisions, and prognosis assessment. Notably, there exist several phenotypical and genotypical differences between Grade II and Grade III gliomas. In this study, we partitioned the entire dataset into two distinct groups based on tumor grades G2 and G3. Subsequently, we conducted a comprehensive analysis of our risk model for each group independently. It’s noteworthy that our study results revealed that the risk models demonstrated good accuracy in both G2 group and G3 group, as shown in Supplementary Figure S3. Specifically, G2 group exhibited higher accuracy in short-term predictions compared to G3 group. Furthermore, there is a consistent expression of risk genes across both groups. This analytical approach has provided a robust framework for evaluating the effectiveness and precision of our risk model in differentiating between the prognoses associated with these two tumor grades.

In the multivariate analysis (p < 0.05), we confirmed that three clinical variables, age, grade, and IDH mutations, were significant prognostic factors for LGG (Figure 8B). However, we wanted to determine whether our risk score model was more accurate than these clinical parameters alone in predicting the survival outcomes at 1-year, 3-year, and 5-year intervals. For this purpose, we conducted further validation of the risk model. Upon validation, we obtained AUC (area under the curve) values for age of 0.572, 0.627, and 0.606 at 1 year, 3 years, and 5 years, respectively (Figure 8C). The AUC values for grade were 0.670, 0.691, and 0.684 at 1 year, 3 years, and 5 years, respectively (Figure 8D). Similarly, for IDH mutations, the AUC values were 0.693, 0.679, and 0.633 at 1 year, 3 years, and 5 years, respectively (Figure 8E). These results indicate that our risk model demonstrated higher AUC values across all time intervals. Based on this comprehensive evaluation, we conclude that the risk score derived from our model is a suitable and independent predictor of prognosis in LGG patients, exhibiting better accuracy than individual clinical parameters such as age, grade, and IDH mutation status.

To predict the survival of LGG patients from a clinical perspective, we utilized the entire dataset to construct a nomogram. The nomogram incorporated age, grade, IDH mutation status, and risk score as variables to predict prognosis (Figure 8F).

Furthermore, we examined the Schoenfeld model residuals plotted against age, grade, IDH mutations, and risk score. These plots suggested that these predictive factors should be incorporated into the model (Figure 8G). As shown in Figure 8H, we observed AUC values of 0.765, 0.798, and 0.760 at one, three, and 5 years, respectively, indicating a strong correlation between the nomogram and observed survival probability.

We also calculated the C-index of our risk score model in the testing set and the entire set, spanning from the first year to the 10th year, yielding C-index values above 0.6 (Figure 8I).

Based on the nomogram scores, we divided the entire set into a high-risk group (n = 526) and a low-risk group (n = 526). Throughout the follow-up period, we performed Kaplan‒Meier survival analysis on the entire set. The results demonstrated a significantly increased OS in the low-risk group compared to that in the high-risk group (p < 0.05) (Figure 8J). To visualize the distribution of each factor in the sample, we assigned values to dichotomous variables (e.g., grade II = 1, grade III = 2, IDHwt = 2, and IDHmut = 3). Figures 8K–M display the distribution of risk factors in a heatmap and the survival status and nomogram scores in the entire set.

These findings underscore the reliability and effectiveness of our immune-related gene prognostic model as well as the trustworthiness of our nomogram model.

### 3.8 Clinical utility of the prognostic risk model

To further evaluate the predictive power of the risk model in LGG patients, we examined the association between the risk factors (risk score and risk genes) from the risk model and the clinical parameters, including age, grade, and IDH mutations. Supplementary Figures S4A–D illustrates that in the wild-type group, the levels of specific factors (KLRC3 and RFXAP) were lower compared to those in the IDH mutant group, whereas MR1 levels and the risk score were higher (p < 0.05).

Furthermore, we observed significant correlations between the values of certain factors (MR1 level and risk score) and age (Supplementary Figures S4E, F). As age decreased, the values of these particular factors decreased (p < 0.05). Additionally, in grade III, the values of certain factors (MR1 level and risk score) were higher than those in grade II (p < 0.05) (Supplementary Figures S4G, H).

These findings highlight the relationships between the risk factors (risk score and risk genes) in the risk model and the clinical parameters, providing further insights into the prognostic value of these factors in LGG patients.

## 4 Discussion

LGG is the most common type of malignant primary tumor in the central nervous system among adults. Despite the effectiveness of various treatments, such as postoperative chemotherapy, radiotherapy, immunotherapy, and targeted therapy, the current management of LGG remains unsatisfactory in many cases. Consequently, there has been a growing interest in identifying reliable prognostic biomarkers for LGG, especially with advancements in gene sequencing technology. The exploration of prognostic biomarkers has revealed several significant factors associated with glioma prognosis. Among these factors, the identification of IDH mutations, O-6-methylguanine-DNA methyltransferase (MGMT) promoter methylation, and 1p/19q codeletions have emerged as crucial indicators. These biomarkers have been found to be closely related to the prognosis and clinical outcomes of gliomas, including LGG. Their identification not only aids in prognostic evaluation but also provides crucial insights into potential therapeutic strategies and precision medicine approaches (Chen et al., 2019). Numerous studies have shown that the immune system is key in the progression and initiation of cancer (Angell and Galon, 2013; Gentles et al., 2015). The immune system, via immune cells, up- or downregulates IRGs to kill cancer cells at certain immune checkpoints (Rooney et al., 2015; Sayour and Mitchell, 2017). IRGs can also be mimicked to help some cancer cells elude destruction (Schreiber et al., 2011; Friedrich et al., 2019). Given its role in cancer progression and prognosis, IRGs are believed to be an important predictor of cancer prognosis. Thus, in combination with ESTIMATE algorithms, we performed a joint analysis of TCGA and CGGA databases to provide novel insights into the role of IRGs in the risk stratification and prognosis of LGG. We also calculated immune scores for each patient and found them to be significantly associated with grade, IDH mutation status and prognosis, which was consistent with the results of a previous study (Xu et al., 2021). IDH mutation status, designated as a biomarker by the World Health Organization (WHO), was assessed using the ESTIMATE method, revealing that it is associated with a comparatively better prognosis in LGG patients. Furthermore, through multivariate Cox regression analysis, we confirmed that IDH mutation status serves as an independent risk factor. Recognizing the impact of ssGSEA immune grouping and immune score on tumor development and prognosis, it is reasonable to consider the immune gene signature as a significant predictor. To predict the prognosis of LGG, we identified prognostic IRGs and utilized them to construct a risk model.

In our study, we observed a significant difference in survival between normal brain samples and those with LGG. To investigate this further, we utilized the GTEx database and TCGA database for differential gene expression analysis. Through this analysis, we identified 8832 DEGs between LGG and normal brain samples. Subsequently, we compared these DEGs with the ImmPort database and identified 412 DEIRGs.

To evaluate our model, we divided the dataset into a training set (TCGA) and a testing set (CGGA). In the training set, we performed univariate Cox regression analysis to identify DEIRGs that significantly correlated with OS. We then employed LASSO and multivariate Cox analysis to further refine the gene selection process, resulting in the inclusion of four PDEIRGs of interest: KLRC3, MR1, PDIA2, and RFXAP. These four candidate genes were used to construct a Cox regression risk model for predicting LGG prognosis.

Overall, our model demonstrated promising performance in accurately differentiating patient survival outcomes. Our risk model exhibited superior accuracy when compared with various molecular subtypes classifications, such as 1p/19q codeletion and DNA methylation. Additionally, when compared to age, grade, and IDH mutation status, our univariate and multivariate Cox regression analyses revealed that our model was a superior independent prognostic factor for LGG patients. The findings convincingly demonstrated the model’s ability to accurately differentiate between distinct survival outcomes for patients.

The KLRC3 gene encodes the NKG2E protein, which was first identified in natural killer (NK) cells. It belongs to the NKG2x family and forms a heterodimeric complex with CD94, which is involved in immune recognition and regulation (Kaiser et al., 2005; Orbelyan et al., 2014). This interaction involves HLA-E, an MHC class Ib protein that is known to play a role in immunosuppressive mechanisms. HLA-E is highly expressed in various tumors, including melanoma, colon cancer, and glioblastoma (Kaiser et al., 2005; Wischhusen et al., 2005; Bianchini et al., 2006; Derre et al., 2006). Cheray et al. (2017) provided evidence supporting the crucial role of KLRC3 in glioblastoma cell invasiveness, as well as its involvement in promoting cancer progression, including glioma radiosensitivity, self-renewal properties, and radioresistance mechanisms. These findings indicate that KLRC3 (NKG2E) has the potential to serve as a novel target for the development of therapeutic strategies against glioblastoma. Recently, a unique subset of T cells known as mucosa-associated invariant T cells (MAIT cells) has garnered significant attention due to their abundance in humans and their involvement in various infectious and noninfectious diseases. MAIT cells possess distinct specificity for microbial riboflavin derivative antigens, which are presented by the major histocompatibility complex (MHC) class I protein MR1. Under normal healthy conditions, the expression of MHC class I-related protein 1 (MR1) on the cell surface is generally low. However, it is upregulated in various disease states or when cells are exposed to microbial antigens, indicating its involvement in immune responses (McWilliam and Villadangos, 2018). The presence of MR1 transcripts detected in brain and kidney tumor foci strongly suggests the involvement of mucosal-associated invariant T (MAIT) cells in tumor immunity (Godfrey et al., 2019). MAIT cells are a subset of proinflammatory innate T cells known to secrete cytotoxic proteins and directly eliminate tumor cells. As a result, they have the potential to serve as effective anticancer effector cells (Gold et al., 2010; Jeffery et al., 2016). MAIT cells have been demonstrated to play both passive and active roles in antitumor immune responses or in conditions that can promote tumor growth (Jeffery et al., 2016; Won et al., 2016). There is limited research on the correlation between protein disulfide isomerase family member 2 (PDIA2) and tumor prognosis. PDIA2 is primarily associated with protein processing and translocation, but previous studies have also suggested its potential involvement in antigen presentation (Walker et al., 2013). However, regulatory factor X-associated protein (RFXAP) is an essential transcription factor for major histocompatibility complex (MHC) class II molecules. It has been demonstrated to downregulate the expression of MHC class II molecules in dendritic cells (DCs) and macrophages, thereby inhibiting CD4^+^ T-cell infiltration (Surmann et al., 2015; Wu et al., 2019). RFXAP has been associated with survival outcomes in various solid tumors. Previous studies propose that the immune system not only aids in fighting early-stage tumors but also promotes tumor outgrowth by exposing the tumor to immune effectors and preventing immunogenicity (Shankaran et al., 2001; Dunn et al., 2002). For instance, pancreatic cancer cells can secrete exosomal microRNA (miRNA/miR)-212-3p, which inhibits RFXAP expression in DCs, thereby suppressing MHC class II expression and promoting immune escape in pancreatic tumors (Ding et al., 2015). These theories provide robust support for the model we constructed.

In order to clarify the specificity of risk genes and risk models for low-grade gliomas (LGG) as compared to GBM, we compared the expression of risk genes in LGG and GBM. The results showed that there were differences in the expression of the four risk genes in LGG and GBM, especially KLRC3 and MR1. KLRC3 is expressed at higher levels in LGG compared to GBM, while MR1 is relatively downregulated in LGG compared to GBM. We believe that we cannot consider the specificity of a single gene expression level in LGG, but should comprehensively consider the specificity of the risk prediction model constructed by four risk genes in LGG. After incorporating the model into GBM data, GBM patients were classified as high-risk groups based on their riskscore scores (Supplementary Figure S5).

To enhance the precision of the model, we developed a nomogram analysis that combines the risk model with additional clinical features (such as age, grade, and IDH mutation status) to predict OS in patients with LGG. The area under the ROC curve (AUC) demonstrated that the nomogram exhibited favorable predictive performance. Consequently, our findings suggest that the nomogram we constructed is a dependable tool for identifying high-risk LGG patients, facilitating early interventions, and assisting in the development of personalized treatments for LGG.

Moreover, we conducted an analysis to explore the correlation between our risk model and specific clinical parameters. Interestingly, we observed that certain factors in our model significantly influenced the progression of LGG. This further strengthens the notion that our model holds significant predictive power in clinical applications. Prior research has shown that alterations in immune infiltrating cells within the tumor microenvironment may be associated with prognosis (McGranahan et al., 2016; Sobral-Leite et al., 2019). Pan et al. (2018) discovered that the interactions between T cells and microglia/macrophages play a crucial role as stromal determinants in supporting the growth of LGG. In our research, there was a positive correlation between the risk score of patients with LGG and the infiltration of immune cells, including B cells, CD4^+^ T cells, CD8^+^ T cells, dendritic cells, macrophages, and neutrophils. These findings further validated our risk model, which not only aids in evaluating the immune microenvironment but also provides guidance for immunotherapy interventions.

Lin et al. (2020) developed a prognostic risk model comprising a signature of five mRNA molecules that has the ability to predict the outcome of pediatric Wilms tumor (WT) patients. This has important implications for comprehending therapeutic targets in pediatric Wilms tumor (WT) patients. Wenli Li et al. developed a dependable prognostic risk model consisting of six genes with the purpose of predicting OS in hepatocellular carcinoma (HCC) patients. The Cox regression model of IRGs constructed by Lin et al. for the prognostic stratification of papillary thyroid cancer can differentiate between patients at high and low risk of mortality (Lin et al., 2019).

We observed that previous research had also developed an IRGs risk model for LGG (Zhang et al., 2020). We verified the formula mentioned in the article and the criteria for dividing into high and low-risk groups, with the specific verification process detailed in the Supplementary Tables S1, S2. Our verification results did not align with the descriptions provided in the paper, leading us to believe that the formula might be missing a part of the calculation process (Supplementary Figure S6). Moreover, despite the article being published in 2020, it utilized data from 2015. Given that TCGA continuously updates its database, including adding and removing numerous sample data, we consider the use of this database to be outdated. Furthermore, this study did not analyze or discuss key prognostic factors for LGG proposed by the WHO, such as IDH status, DNA methylation, and 1p/19q codeletion, nor did it demonstrate the superiority of its model relative to these factors. The expression levels of the risk genes in question were also not experimentally validated.

Compared to previous studies, our research had several distinguishing features. In terms of data selection, we utilized a combination of transcriptome data from LGG tissues in the TCGA database and normal brain tissue transcriptome data from the GTEx database. To ensure accuracy and reliability, we employed the TCGA database as the training set to construct the model and then validated it using an external testing set (the CGGA database). Furthermore, we employed various algorithms, such as univariate Cox, multivariate Cox, and Lasso regression, to identify IRGs for inclusion in our model. Notably, our model’s predictive superiority exceeds the prognostic factors for LGG proposed by WHO, such as IDH status, DNA methylation, and 1p/19q codeletion. We also conducted validation of the risk genes involved in the risk model in cell lines. As a result, our model demonstrated superior predictive capabilities for the development of LGG compared to previous models.

Biomarkers often present a balance between specificity and sensitivity, leading to potential false positives or negatives in certain contexts. Future research could enhance diagnostic accuracy by increasing sample sizes, incorporating additional validation cohorts, or employing multimarker panels. While biomarkers may show strong predictive values in specific ethnicities or populations, their applicability can vary across different groups. This necessitates designing future studies to include diversity and representativeness, validating and improving biomarker universality through large-scale, multicenter studies across different ethnicities and regions. Biomarker detection generally requires specialized techniques and equipment, which may limit their use in low-resource settings. To address this, the development of more economical and user-friendly detection methods, or the adaptation and optimization of existing, lower-cost technologies, is crucial. Even if some biomarkers are statistically associated with disease states, their biological mechanisms and clinical relevance might remain unclear. Future research should focus on exploring the mechanisms linking biomarkers with disease onset and progression, and on integrating these biomarkers effectively into clinical decision-making processes. Biomarker research often involves sensitive personal information and biological samples, necessitating stringent ethical review and privacy protection for participants. Future studies must adhere to rigorous ethical standards to ensure the security of personal information and the protection of participant rights.

While this study has yielded some interesting findings, we must honestly acknowledge several limitations. Firstly, all data were sourced from public databases. Although we conducted some validation in cell lines, the lack of *in vivo* experimental data remains a significant gap. Future research endeavors should aim to validate our findings further using clinical samples and animal model data, expanding the sample size, particularly by incorporating multi-center clinical data to enhance the external validity of the study. Secondly, given the continuous evolution of bioinformatics tools, future investigations could leverage more advanced data integration and analysis methods to deepen our understanding of LGG heterogeneity. Utilizing single-cell sequencing techniques may unveil genetic variations and expression differences among different cell subpopulations within tumors, thereby providing better insights into the pathogenesis of LGG. Furthermore, our study only focused on static genomic information, and the impact of dynamic epigenetic regulation and environmental factors has not been thoroughly investigated. Future studies could comprehensively explore the pathogenesis of LGG by integrating epigenetics and environmental exposure data. Research into treatment avenues is also an important direction for future inquiry. Integrating genomic data with clinical treatment outcomes could facilitate more in-depth bioinformatics analyses to identify patient subgroups that may respond favorably to specific treatment regimens.

## 5 Conclusion

In summary, this study presents a four-gene model that shows promise as a reliable tool for predicting the prognosis of patients with LGG. The risk score generated by the model demonstrated its potential as an independent prognostic marker. The nomogram developed based on the model can be instrumental in guiding personalized treatment decisions for patients with LGG in clinical practice. Moving forward, future research efforts can focus on employing more advanced and rational bioinformatics strategies to further refine and enhance the predictive accuracy of the model. This may involve incorporating additional molecular markers, genetic variants, or other relevant factors to improve the prognostic capacity of the model. Overall, this study represents a valuable step toward developing a robust prognostic tool for LGG patients, opening avenues for personalized treatment approaches. Continued research and validation will be crucial to establish the clinical utility and applicability of the four-gene model in routine clinical practice.

## Data Availability

The original contributions presented in the study are included in the article/Supplementary Material, further inquiries can be directed to the corresponding author.
