# Supplementary material for: Prognostic value of four immune-related genes in lower-grade gliomas: a biomarker discovery study
Source: Front Genet. 2024 Aug 12;15:1403587. doi: 10.3389/fgene.2024.1403587 (PMC11347950; doi:10.3389/fgene.2024.1403587)
Supplement: Supplementary file 3 [file DataSheet1.docx]

Supplementary Material

# Supplementary Figures and Tables

## Supplementary Figures


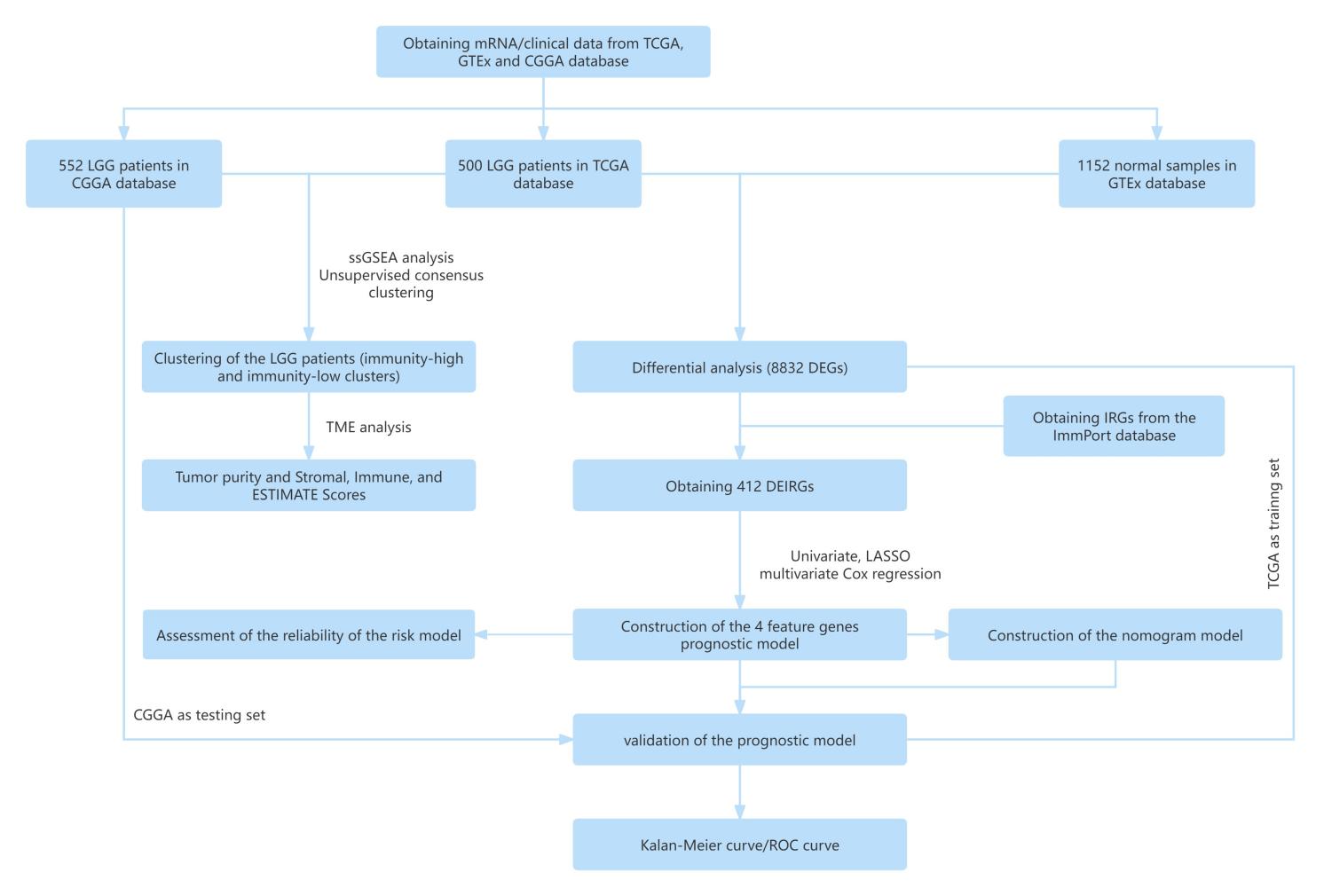
**Supplementary Figure Graphical Abstract**


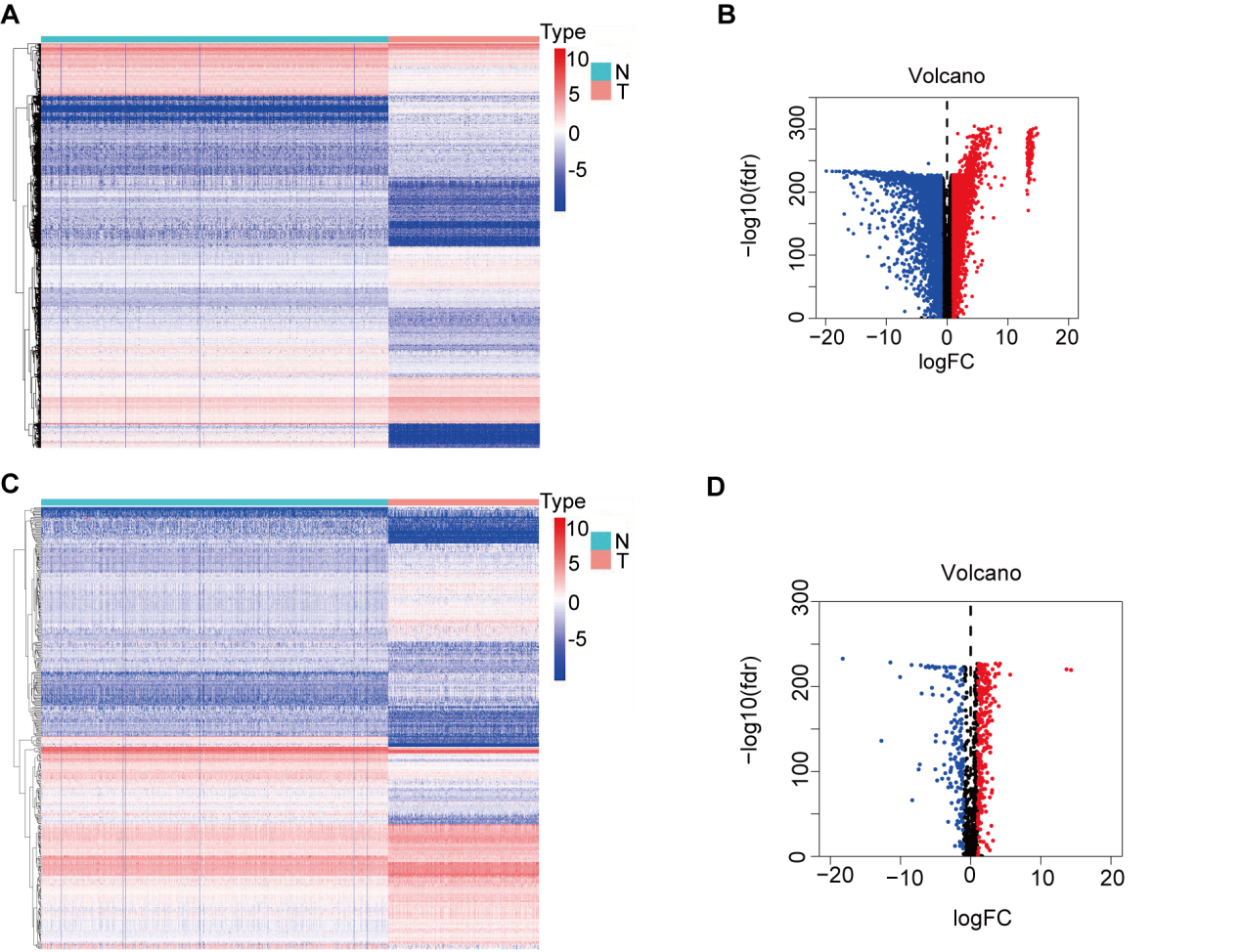


**Supplementary Figure 1.** Expression of DEGs and DEIRGs in the two sample groups. (A) Heat map of DEGs; the blue to red spectrum indicates low to high gene expression. (B) Volcano plot of DEGs; the blue dots represent down-regulated DEGs, the red dots represent up-regulated DEGs and the black dots represent DEGs that were not significantly differentially expressed. (C) Heat map of DEIRGs; the blue to red spectrum indicates low to high gene expression. (D) Volcano plot of DEIRGs; the blue dots represent down-regulated DEIRGs, the red dots represent up-regulated DEIRGs and the black dots represent DEIRGs that were not significantly.


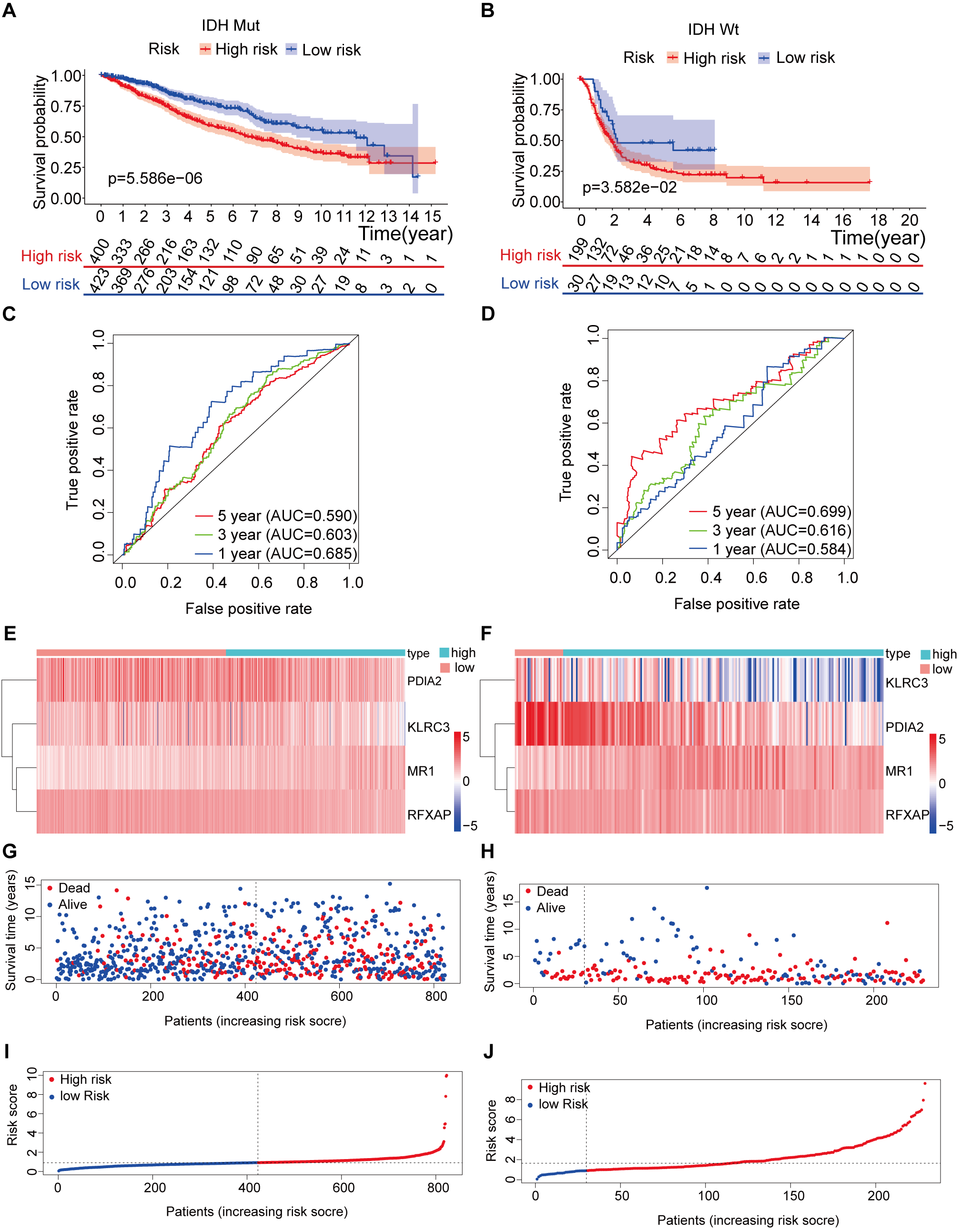


**Supplementary Figure 2.** Validation of the immune-related gene prognostic signature in the IDH mutant and IDH wild-type groups. (A-B) The survival status of patients in the high-risk and low-risk groups in the IDH mutant and IDH wild-type groups. (C-D) The prognostic signature’s time-independent ROC curve at 1-, 3-, and 5-years in the IDH mutant and IDH wild-type groups. (E-F) Expression patterns of risk genes in the IDH mutant and IDH wild-type groups. (G-H) A scatter plot depicts the survival of LGG samples in the IDH mutant and IDH wild-type groups. (I-J) Each LGG sample’s risk curve is reordered by the risk score in the IDH mutant and IDH wild-type groups.


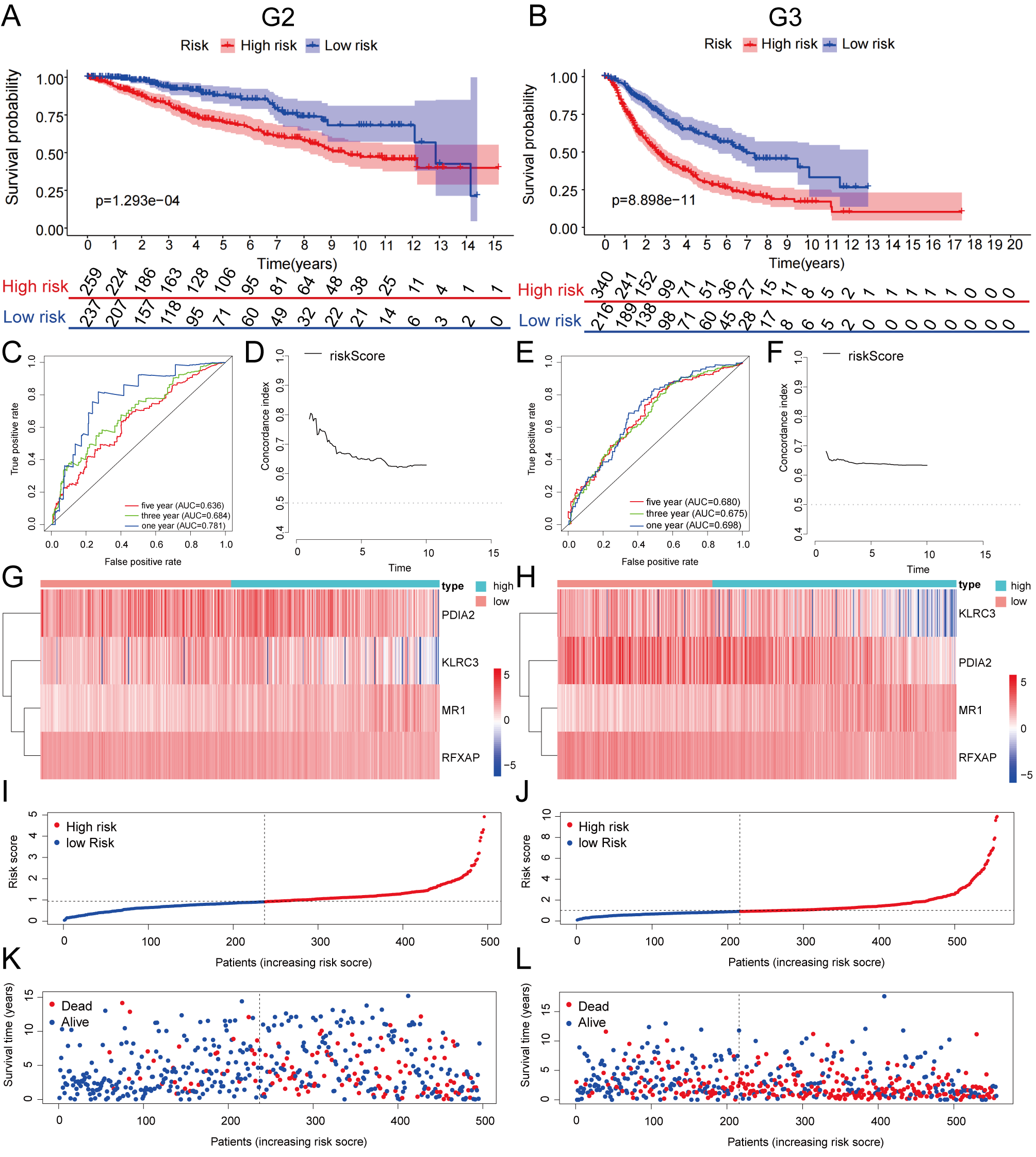


**Supplementary Figure 3.** Validation of the immune-related gene prognostic signature in the G2 and G3 groups. (A-B) The survival status of patients in the high-risk and low-risk groups in the G2 and G3 groups. (C-F) The prognostic signature’s ROC curve and C-index in the G2 and G3 groups. (G-H) Expression patterns of risk genes in the G2 and G3 groups. (I-J) A scatter plot depicts the survival of LGG samples in the G2 and G3 groups. (K-L) Each LGG sample’s risk curve is reordered by the risk score in the G2 and G3 groups.


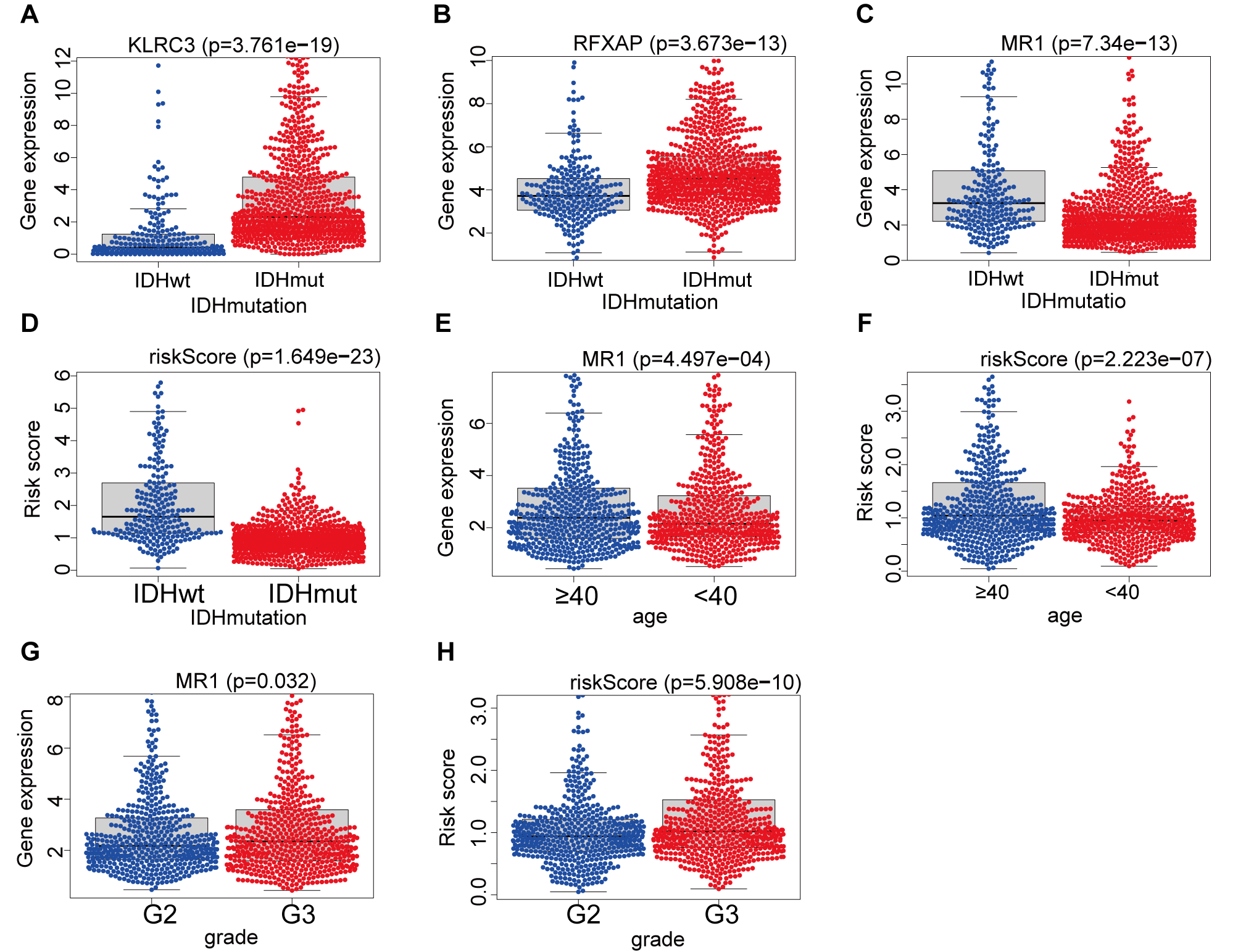


**Supplementary Figure 4.** Relationships of the variables in the model with the clinical characteristics of patients in the entire set (p<0.05). (A) KLRC3 expression and IDH mutation. (B) RFXAP expression and IDH mutation. (C) MR1 expression and IDH mutation. (D) Risk score and IDH mutation. (E) MR1 expression and age. (F) Risk score expression and age. (G) MR1 expression and grade. (H) Risk score expression and grade.


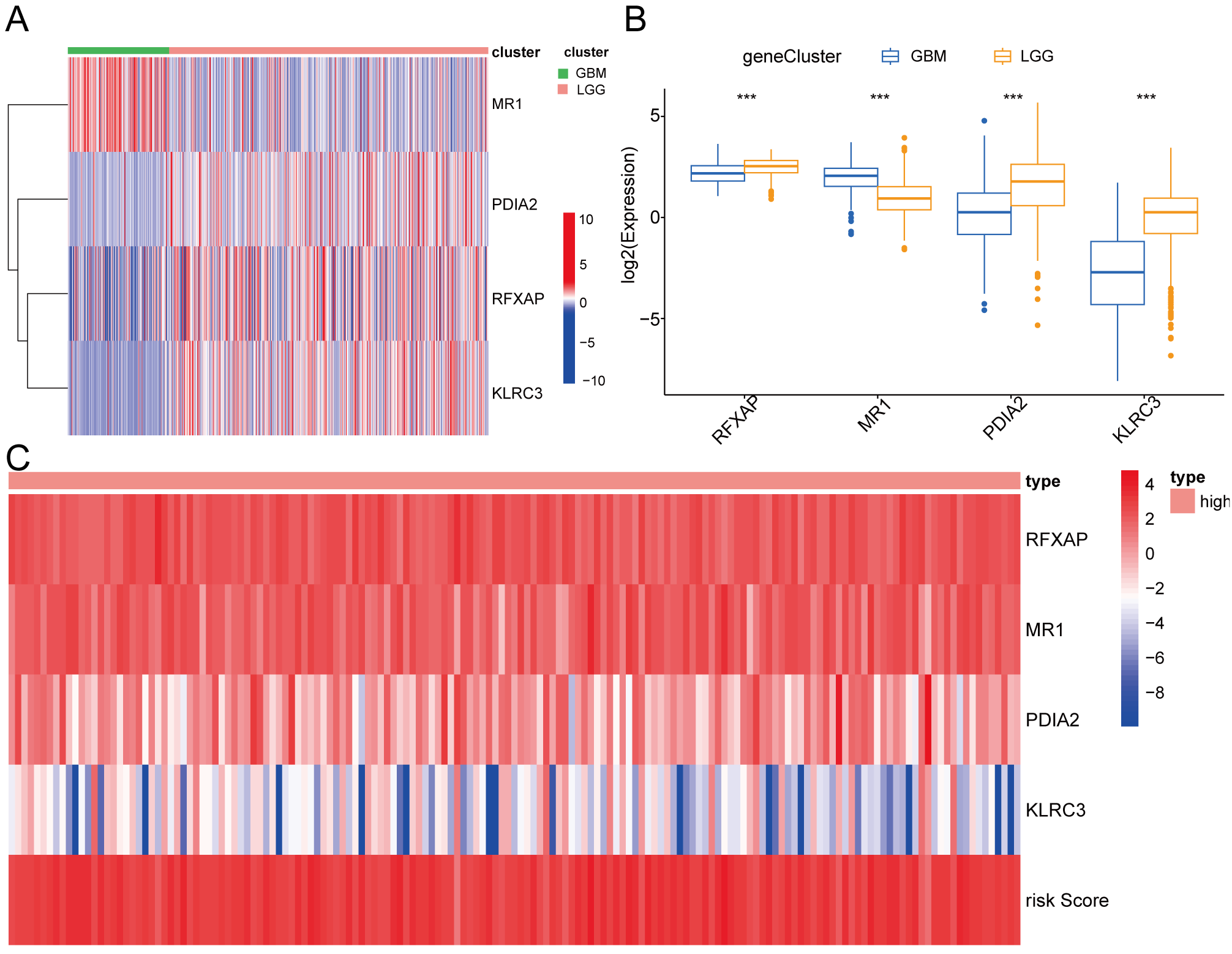


**Supplementary Figure 5.** Expression of risk genes in LGG and GBM. (A and B) Expression patterns of risk genes in patients with LGG and GBM. (C) Expression patterns of risk genes and risk scores in the high-risk group of GBM.


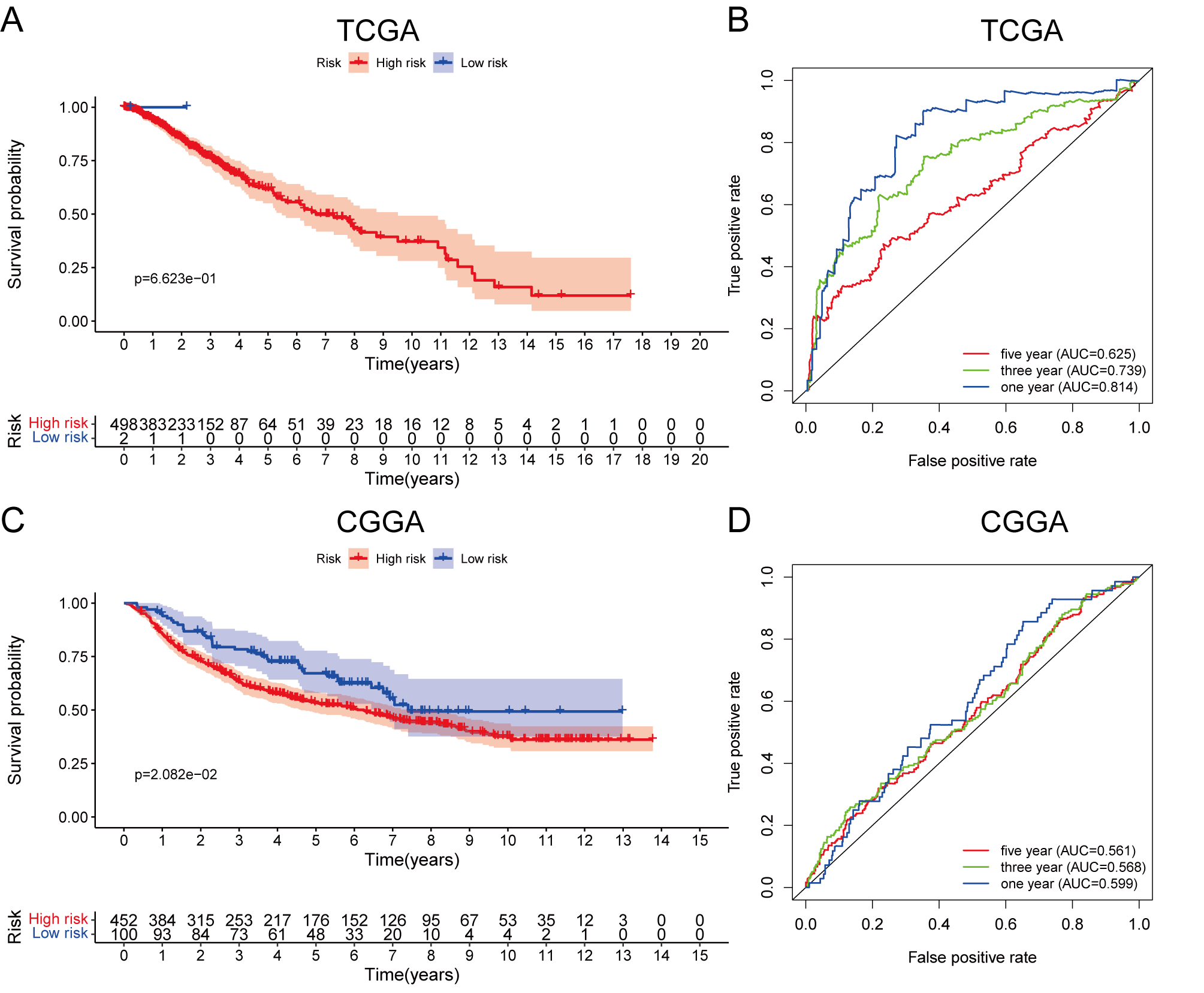


**Supplementary Figure 6.** Validation of a Risk Model from Prior Research Studies. (A) The survival status of patients in the TCGA database. (B) The prognostic signature’s ROC curve in the TCGA database. (C) The survival status of patients in the CGGA database. (D) The prognostic signature’s ROC curve in the CGGA database.
